# *Cucumeropsis mannii* seed oil ameliorates Bisphenol‐A‐induced adipokines dysfunctions and dyslipidemia

**DOI:** 10.1002/fsn3.3271

**Published:** 2023-02-18

**Authors:** Patrick M. Aja, Chukwu D. Chiadikaobi, Peter C. Agu, Boniface A. Ale, Onyedika G. Ani, Ezebuilo U. Ekpono, Hilary A. Ogwoni, Joshua N. Awoke, Patience N. Ogbu, Lucy Aja, Felix E. Nwite, Oliver U. Ukachi, Obasi U. Orji, Peter C. Nweke, Chinedu O. Egwu, Ejike U. Ekpono, Gift O. Ewa, Ikechuku O. Igwenyi, Deusdedit Tusubira, Christian E. Offor, Ekenechukwu K. Maduagwuna, Esther U. Alum, Daniel E. Uti, Amobichukwu Njoku, Victor A. Atoki, Chinaza G. Awuchi

**Affiliations:** ^1^ Department of Biochemistry Ebonyi State University Abakaliki Nigeria; ^2^ Department of Biochemistry Mbarara University of Science and Technology (MUST) Mbarara Uganda; ^3^ Department of Biochemistry Kampala International University Bushenyi Uganda; ^4^ Department of Biochemistry University of Nigeria Nsukka Nigeria; ^5^ Department of Public Health and Nutrition University of Chester Chester UK; ^6^ Department of Medical Biochemistry Alex‐Ekwueme Federal University, Ndufu‐Alike, Ikwo Abakaliki Ebonyi State Nigeria; ^7^ Department of Science Education Ebonyi State University Abakaliki Nigeria; ^8^ Department of Biochemistry Federal University of Health Sciences Otukpo Nigeria; ^9^ Department of Science Laboratory Technology Federal Polytechnic Oko Oko Anambra State Nigeria; ^10^ School of Natural and Applied Sciences, Kampala International University Kampala Uganda

**Keywords:** adipokines, bisphenolA, *Cucumeropsis mannii* seed oil, dyslipidemia, natural products

## Abstract

This study demonstrated the therapeutic potentials of *Cucumeropsis mannii* seed oil (CMSO) capable of alleviating BPA‐induced dyslipidemia and adipokine dysfunction. In this study, we evaluated the effects of CMSO on adipokine dysfunctions and dyslipidemia in bisphenol‐A (BPA)‐induced male Wistar rats. Six‐week‐old 36 albino rats of 100–200 g weight were assigned randomly to six groups, which received varied doses of BPA and/or CMSO. The administration of BPA and CMSO was done at the same time for 42 days by oral intubation. The adipokine levels and lipid profile were measured in adipose tissue and plasma using standard methods. BPA induced significant (*p* < .05) increases in triglycerides, cholesterol, leptin, LDL‐C, and atherogenic and coronary risk indices in adipose tissue and plasma, as well as a decrease in adiponectin and HDL‐C levels in Group II animals. BPA administration significantly (*p* < .05) elevated Leptin levels and reduced adiponectin levels. BPA plus CMSO reduced triglycerides, cholesterol, leptin, LDL‐C, and atherogenic and coronary risk indices while increasing adiponectin levels and HDL‐C in adipose tissue and plasma (*p* < .05). The results showed that BPA exposure increased adipose tissue as well as serum levels of the atherogenic index, triglycerides, cholesterol, coronary risk index, LDL‐C, leptin, and body weight with decreased adiponectin levels and HDL‐C. Treatment with CMSO reduced the toxicities caused by BPA in rats by modulating the body weight, adiponectin/leptin levels, and lipid profiles in serum and adipose tissue. This study has shown that CMSO ameliorates BPA‐induced dyslipidemia and adipokine dysfunctions. We suggest for further clinical trial to establish the clinical applications.

## Graphical abstract

BPA plus CMSO reduced triglycerides, cholesterol, leptin, LDL‐C, and atherogenic and coronary risk indices while increasing adiponectin levels and HDL‐C in adipose tissue and plasma (*p* < .05). Treatment with CMSO reduced the toxicities caused by BPA in rats by modulating the body weight, adiponectin and leptin levels, and lipid profiles in adipose tissue and serum.

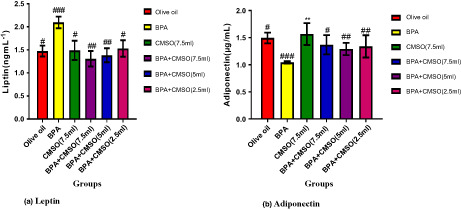

## 1 INTRODUCTION

Endocrine disruptors (Eds or EDCs) are substances that work by imitating endogenous hormones, disrupting the signaling system, and causing a slew of metabolic problems (Flint et al., 2012; Metz, 2016). 2, 2‐Bis (4‐hydroxy‐phenyl) propane shortened as BPA is among the common xenobiotics acting as endocrine disruptors (Awuchi & Awuchi, 2019; Gabr et al., 2017). The major pathways of BPA human exposure have been identified as ingestion, inhalation, and skin contact (Talpade et al., 2018). Increased temperature, extended storage duration, repetitive use, and basic/acidic foods in bottles/cans have all been linked to cancer (Adeghe & Emejulu, 2016). Though, leached bisphenol A hydrolyzes in drinks/food, dietary exposure is the major human exposure, environmental contamination, among other things, may increase BPA exposure (Adeghe & Emejulu, 2016; Stragierowicz, 2015). Due to the production of peptide hormones called adipokines, including adiponectin and leptin, the adipose tissues play crucial roles in the biological system's endocrine system. These adipokines act either systemically, in the form of endocrine action, or locally, in the form of paracrine/autocrine action, communicating information on triacylglycerides (TAG) (adipose tissue's energy reserves) to the brain and other tissues (Nelson & Cox, 2008). Furthermore, in animals, the adipose tissue serves as a primary fuel depot by storing TAGs and regulating thermal homeostasis (Tusubira et al., n.d.; Müllerová & Kopecký, 2007).

Like many endocrine disruptors, bisphenol A is lipophilic and has a long half‐life, and can bioaccumulate in the adipose tissues resulting in weight gain through adipogenesis stimulation (Akash et al., 2020). The action of BPA on adipose tissue implies that it is an obesogen, causing aberrant fat accumulation and obesity (Apau et al., 2018; Ariemma et al., 2016). Bisphenol A exposure also interferes with the functions of adipokines in adipose tissues (Ariemma et al., 2016; Rönn et al., 2014).

Plants provide man with a variety of structurally unique natural products, which offer a variety of therapeutic and nutraceutical potentials and contribute to the enhancement of human's health (Watal et al., 2014). The gourd family, also called Cucurbitaceae, is among the most genetically enriched restorative medicinal plants, as well as a source of fats, protein, and other important nutrients in diets (Desai et al., 2018; Dhakad et al., 2017). In moist, humid climates, especially in the regions of Cameroon and southwestern Nigeria, the nonhardy legume *Cucurbitaceae mannii* (*C. mannii*), belonging to the family Cucurbitaceae, grows as a tendril creeper/climber (Watal et al., 2014). (Adeghe & Emejulu, 2016) stated that in Nigeria, this plant is also known as “Agushi” in Hausa, “Egusi” in Yoruba, and “Egwusi” in Igbo. It is also known as white‐seed melon and *cucumeropsis Manni* in English (Adeghe & Emejulu, 2016).

Dyslipidemia is prevalent among patients with coexisting risk factors of cardiovascular diseases, including diabetes, diabetes, hypertension, human immunodeficiency virus, etc. (Gebreegziabiher et al., 2021; Tufail et al., 2021; Yasmin et al., 2021). It is associated with over 50% of cases of ischemic heart disease worldwide, and over 4 million deaths per year (Gebreegziabiher et al., 2021). The prevalence of dyslipidemia in African adult population alone is estimated at over 25.5% (Gebreegziabiher et al., 2021). Adipose tissue dysfunctions caused by many factors, such as obesity, lead to dysregulated production of adipokine, which has systemic and local effects on inflammatory cells (Kim & Choi, 2020). Imbalance in adipokine results in the development of chronic inflammation that plays a role in the development of cardiovascular and metabolic diseases (Kim & Choi, 2020).

According to research, CMSO could be suitable edible oil for lowering cardiovascular diseases and has been found to inhibit phosphodiesterase–5 and arginase, suggesting that it has erection‐inducing qualities (Amin et al., 2018). Studies reported that CMSO reduces alterations in the histology and biochemistry of testicles against BPA‐induced toxicity (Agu et al., 2022). *Cucumeropsis mannii* contains 31.4% crude protein and all the essential amino acids, and 52.5% total fat in its seeds with abundant fatty acids such as oleic (15.90%), linoleic (62.42%), stearic (10.26%), palmitic (10.27%), etc. (Besong et al., 2011), as well as fiber, carbohydrates, and other important compounds, including bioactive compounds such as polyphenols, alkaloids, etc, all of which have been shown to exert important biological properties in various studies (Khalid et al., 2022; Ofoedu et al., 2022; Rahim et al., 2022). As a result, this study aims to explore if *C. mannii* seed oil could help maintain systemic lipid homeostasis while also reducing the harmful effects of BPA in adipose tissue. In this study, we evaluated CMSO effects on in BPA‐induced adipokine dysfunctions and dyslipidemia using male Wistar rats. The study showed the therapeutic potentials of CMSO capable of alleviating BPA‐induced dyslipidemia and adipokine dysfunction.

## 2 MATERIALS AND METHODS

### 2.1 Chemicals

Pure pellets BPA [2,2‐bis(4‐hydroxyphenyl) propane] and the chemical reagents for the study were all analytical grades and obtained through Bristol Scientific from Sigma Aldrich Company, United Kingdom.

### 2.2 Collecting and authenticating plants

*Cucumeropsis mannii* Naud seed was used in this study, and it was acquired from Izzi LGA, Nigeria. The obtained plant was identified by Mr. Nwankwo, E.O., a taxonomist at Ebonyi State University, Nigeria (EBSU). For the purpose of reference, a portion of the *C. mannii* seed was maintained at the herbarium of EBSU with voucher Reference number: EBSU‐H‐396.

### 2.3 CMSO extraction

CMSO was extracted by applying the traditional and mechanical methods of Kate et al. (2014) and modified by Agu et al. (2022). During extraction, warm water drops were added to improve oil (Colucci et al., 2020; Nwozo et al., 2023). The water helps in the rupturing of the cells, partly by binding to gum/mucilage (hydrocolloids) (Dror et al., 2006), which are allowed to sediment. The extracts were stood for 5–7 days undisturbed to sediment and were then separated by decantation to recover oil with purer quality. The oil was then kept in clean bottles for use.

### 2.4 Test for acute toxicity

*CMSO* acute toxicity in Wistar Albino rats (male) was evaluated according to OECD (2008) as specified in guideline No. 425. Acute toxicity was done using the test procedure of limit dose according to the guideline No. 425. For this experiment, 2 months old albino Wistar rats (male) were employed for the animal study and were acclimatized in the laboratory for 7 days before the experiment. Fifty ml/kg CSMO was orally given to a female rat after fasting overnight. Thereafter, the rat was strictly monitored for any observed behavioral or physical change for an initial 30 min after the extract's administration, and then periodically observed for the next 24 h, with more attention within the first 4 h, and then monitored on daily basis for 14 days (Agu et al., 2022; Eleazu et al., 2021). Foods were given to the rats after 3‐ to 4 h administration of CMSO. After the first rat survival, the other four rats (male) were recruited, then fasted for 4 h. The rats subsequently received the same CMSO dose followed by the same observation/monitoring, which continued for additional 14 days to watch for possible toxicity (Agu et al., 2022; Eleazu et al., 2021). At 50 ml/kg limit test dose, there were no signs of gross behavioral or physical changes in the rats, such as motor activity, reduction in feeding, or hair erection during the 24 h and 14‐day monitoring periods. Consequently, 5 ml/kg limit dose (10%) was chosen as the intermediate/middle dose, 2.5 ml/kg (half of this) was chosen as the lower dose, while 7.5 ml/kg (1.5 times the middle dose) was chosen as the higher dose according to the guideline of OECD (2008) as stated in the OECD guideline No. 425.

### 2.5 Experimental rats

The animals used for the experiment were albino Wistar rats obtained from the Animal House, University of Nigeria, Nsukka, Nigeria. They were obtained and put in rat cage made of stainless steel in the animal house that was well ventilated at the Department of Biochemistry, EBSU. The rats were allowed 7 day acclimatization under good sanitary/laboratory conditions at ambient temperature and a dark/light cycle of 12 h (Messaoudi et al., 2022; Rahim et al., 2023). They were allowed unrestricted access to Vital Feed® and water. The rats were well handled as recommended/approved by the Ethical Committee, with EBSU/BCH/ET/20/003 ethical approval number.

### 2.6 BPA dissolution

The BPA pellets were ground. The solution of BPA was formed by dissolving 5 g BPA in 100 ml olive oil according to Alboghobeish et al. (2019). Olive oil served as the vehicle and was administered to the normal control.

### 2.7 Experimental design

The study made use of 36 male rats (6 weeks) of 100–200 g weight, which were randomly grouped into six groups labeled groups I to VI, with each group having six rats. Groups I to III were control, whereas groups IV to VI were treated groups. Groups I, II, and III were given 1 ml olive oil, 100 mg/kg bw BPA, and 7.5 ml/kg bw CMSO, respectively. Groups IV, V, and VI received 100 mg/kg bw BPA + 7.5 mg/kg bw CMSO, 5 mg/kg bw CMSO, and 2.5 mg/kg bw CMSO, respectively. Body weight was measured every 7 days during the trials. The CMSO and the BPA were simultaneously administered using oral intubation for 6 weeks, and done once per day (Rahim et al., 2023; Zheng et al., 2020).

### 2.8 Collection of tissue sample

The sacrifice of the animals was done under light anesthesia using cervical dislocation the following overnight fast at the end of the study. Plain sample bottles were used to collect blood through the femoral vein. The plasma was cautiously pipetted into appropriate tubes with labels for examination after centrifuging the blood samples for 5 min at 3000 rpm. The rats' adipose tissues were removed, cleaned from superficial connective tissue, and then kept in a solution of 1:5 v/v ice‐cold 0.25 M sucrose before commencing the analysis.

### 2.9 Determination of biochemical parameters

#### 2.9.1 Lipid profile determination

The Allain and Roschlaw (1979) method was used to determine the total cholesterol. The cholesterol concentration (mg/dl) in samples = sample absorbance/standard absorbance × standard concentration. The concentration of HDL cholesterol was measured according to the Allain and Roschlaw (1979) centrifugation method. The mg/dl HDL cholesterol = sample absorbance/standard absorbance × standard concentration. Triglyceride (TG) concentration was evaluated using Tietz (1990) description. Concentration of TG = sample absorbance/standard absorbance × standard concentration. The concentration of LDL cholesterol was measured using the Friedewald et al. (1972) equation method. LDL cholesterol concentration (mg/dl) = Total cholesterol−(HDL + triglycerides).

#### 2.9.2 Atherogenic index (AI) and coronary risk index (CRI)

The atherogenic index was calculated as described by Eleazu et al. (2018). Atherogenic index = LDL − HDL. The CRI was calculated according to Alladi and Shanmugasundaram (1989) and Abbott et al. (1998): CRI = total cholesterol/HDL.

#### 2.9.3 Determination of leptin and adiponectin levels

The ELISA described by Hotta et al. (2000) was used to measure the plasma leptin level and adiponectin level.

### 2.10 Statistics

The statistical analyses were done with GraphPad Prism 5.04. The data/results were presented as mean standard deviation. anova (One‐way) with Brown–Forsythe test was done. Generally, *p* < .05 (significance level).

## 3 RESULTS AND DISCUSSION

### 3.1 CMSO effects on rats' body weight in BPA‐induced dyslipidemia

**FIGURE 1:**
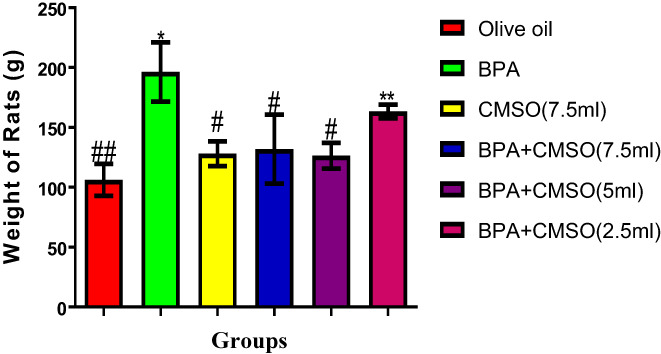
*Cucumeropsis mannii* seed oil effects on body weight on dyslipidemia induced by BPA in rats. The values of the results are presented as mean ± SD (*n* = 6). Mean having different signs have significant difference (*p* < .05).

BPA administration significantly (*p* < .05) elevated the rat weights (Figure 1). However, there was a significant (*p* < .05) decrease in the body weight of rats after coadministration of BPA + CSMO (Figure 1).

### 3.2 CMSO effects on serum lipid profile in BPA‐induced dyslipidemia

**FIGURE 2:**
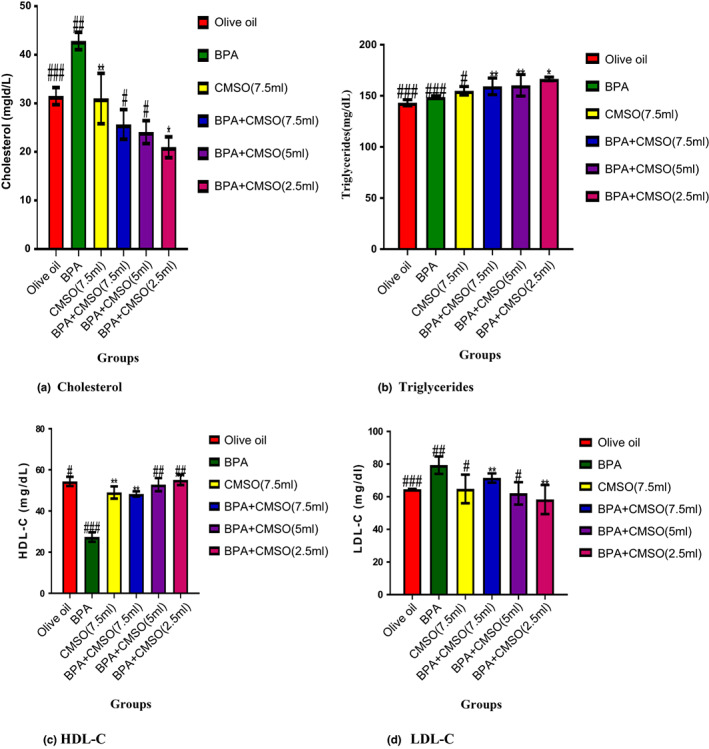
(a–d) Effects of *Cucumeropsis mannii* seed oil on serum lipid profile in dyslipidemia induced by BPA in rats. The values of the results are presented as mean ± SD (*n* = 6). Mean having different signs have significant difference (*p* < .05).

In Figure 2a–d, the result showed that BPA administration significantly (*p* < .05) elevated the serum levels of cholesterol and LDL‐C with a reduction in HDL‐C levels. However, BPA and CMSO coadministration significantly (*p* < .05) reduced cholesterol, triglycerides, and LDL‐C with an elevation of HDL‐C. A nonsignificant(*p* > .05) increase was observed in the level of triglycerides in normal control when compared with the BPA control. But coadministration of BPA and CMSO significantly(*p* < .05) elevated the triglycerides level in serum (Figure 2a–d).

### 3.3 CMSO effects on adipose tissue lipid profile in BPA‐induced dyslipidemia

**FIGURE 3:**
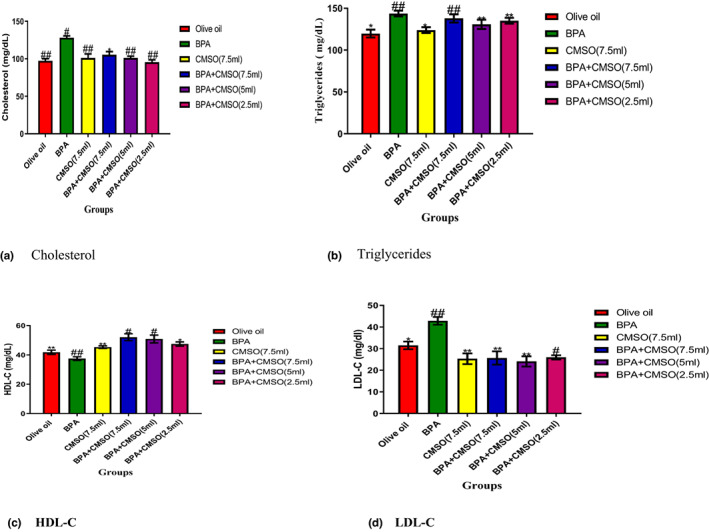
(a–d) *Cucumeropsis mannii* seed oil effects on the lipid profile of adipose tissue in dyslipidemia induced by BPA in rats. The values of the results are presented as mean ± SD (*n* = 6). Mean having different signs have significant difference (*p* < .05).

BPA administration significantly (*p* < .05) elevated the levels of cholesterol, triglycerides, and LDL‐C with a reduction in HDL‐C level in rat adipose tissue. However, BPA and CMSO coadministration significantly (*p* < .05) reduced cholesterol, triglycerides, and LDL‐C with the elevation of HDL‐C (Figure 3a–d).

### 3.4 CMSO effects on CRI in BPA‐induced dyslipidemia in rats

**FIGURE 4:**
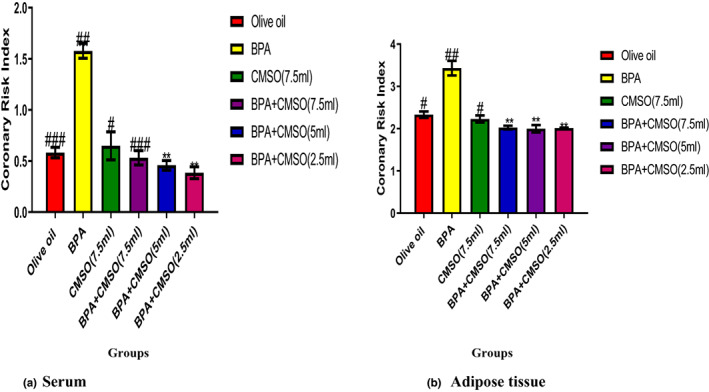
(a,b) *Cucumeropsis mannii* seed oil effects on coronary risk index in dyslipidemia induced by BPA in rats. The values of the results are presented as mean ± SD (*n* = 6). Mean having different signs have significant difference (*p* < .05).

BPA administration significantly (*p* < .05) elevated the CRI in serum and adipose tissue of male rats (Figure 4). BPA and CMSO coadministration in male rats significantly (*p* < .05) reduced the CRI in both adipose tissue and serum of rats (Figure 4a,b).

### 3.5 CMSO effects on atherogenic risk index in BPA‐induced dyslipidemia

**FIGURE 5:**
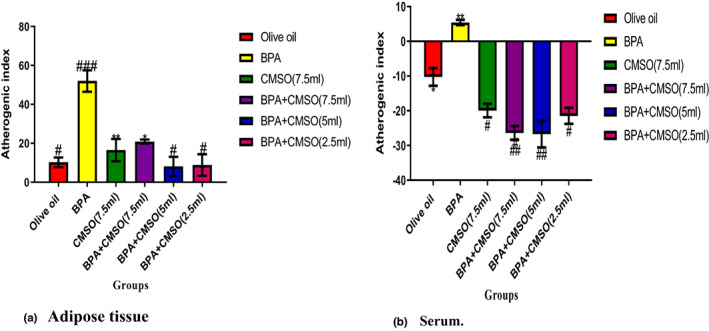
(a,b) *Cucumeropsis mannii* seed oil effects on atherogenic risk index in dyslipidemia induced by BPA in rats. The values of the results are presented as mean ± SD (*n* = 6). Mean having different signs have significant difference (*p* < .05).

BPA administration significantly (*p* < .05) elevated the ARI in adipose tissue and serum of male rats (Figure 5). BPA and CMSO coadministration in male rats significantly (*p* < .05) reduced the ARI in both adipose tissue and serum of rats (Figure 5a,b).

### 3.6 CMSO effects on adipose tissue adipokines in BPA‐induced adipokine dysfunction

**FIGURE 6:**
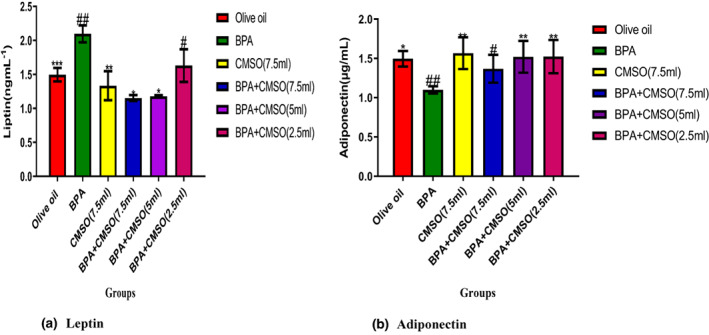
(a,b) *Cucumeropsis mannii* seed oil effects on adipose tissue adipokines in rats induced by BPA‐mediated adipokine dysfunction. The values of the results are presented as mean ± SD (*n* = 6). Mean having different signs have significant difference (*p* < .05).

BPA administration significantly (*p* < .05) elevated leptin level and reduced adiponectin level in adipose tissues of male rats (Figure 6). BPA and CMSO coadministration in male rats significantly (*p* < .05) reduced the level of leptin but elevated the adiponectin level in male rats (Figure 6).

### 3.7 CMSO effects on serum adipokines in BPA‐induced adipokine dysfunction

**FIGURE 7:**
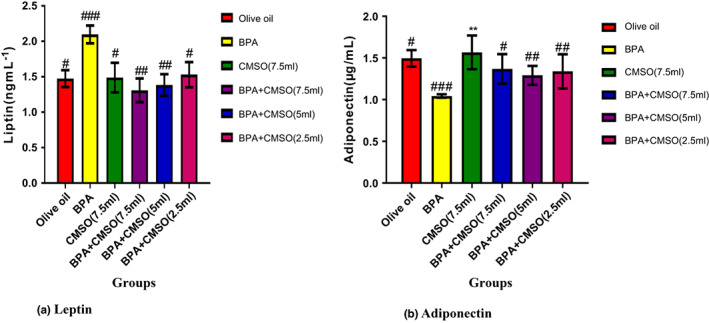
(a,b) *Cucumeropsis mannii* seed oil effects on serum adipokines in rats induced with adipokine dysfunction mediated BPA. The values of the results are presented as mean ± SD (*n* = 6). Mean having different signs have significant difference (*p* < .05).

BPA administration significantly (*p* < .05) elevated leptin levels and reduced adiponectin levels in male rats' serum (Figure 7). BPA and CMSO coadministration in male rats significantly (*p* < .05) reduced the level of leptin though not in a dose‐dependent manner and elevated the adiponectin level in male rats (Figure 7).

## 4 DISCUSSION

In the current study, BPA significantly elevated the body weight of the rats. Gurmeet et al. (2014) found that following BPA exposure at 1, 5, and 100 mg/kg body weight showed no significant difference as compared to the control group. (Miao et al., 2015) found a substantial reduction in body weight and testicular volume in rats exposed to 400 mg/ kg bw of BPA when compared to a control group. Awuchi & Awuchi (2019) described the endocrine disruptive effects of BPA and other endocrine disruptors found in plastic packages. Further studies showed that when male rats were given a modest dose (100 mg/kg bw) of BPA, their body weight did not alter significantly (Nanjappa et al., 2012; Norazit et al., 2012). Our results showed that BPA intoxication increased the body weight of the experimental rats. Neier et al. (2019) reported a gradual increase in body weight in the offspring of 12–27‐weeks ‐old C57BL/6J female mice that were fed on Chow diet Gestation day 9 to PND 21 at a dose of 50 μg/kg/day. According to Tian et al. (2021) reported that exposure to BPA and tetrabromobisphenol A at 20, 100, and 500 μg/L/day elevated the body weight, length, and food intake in male zebrafish. Taylor et al. (2018) reported increased body weight of prenatal mice exposed to 500 mg/kg bw/day. However, CMSO cotreatment reduced the body weight of rats considerably which might be attributed to CMSO's therapeutic efficacy and protective capacity.

Furthermore, our findings showed that BPA treatment substantially increased blood and adipose tissue cholesterol, triglycerides, and LDL‐C levels while decreasing HDL‐C levels in rats. Wang et al. (2020) reported that BPA exposure increased low‐density lipoprotein cholesterol (LDL‐C), triglyceride (TAG), and total cholesterol (TC), with a reduction in high‐density lipoprotein cholesterol (HDL‐C). Similarly, the current study supports a previous study by Zhu et al. (2015) that found BPA to cause a detrimental decrease in total cholesterol (TC), high‐density lipoprotein cholesterol (HDL‐C), as well as a rapid increase in low‐density lipoprotein cholesterol (LDL‐C), triglycerides (TG), and free fatty acids (FFA), altering the HDL‐C transport mechanism.

A study by Cai et al. (2015) also found that BPA raised the levels of FFA in the blood as well as the levels of low‐density lipoprotein (LDL‐C) and triglycerides (TGs) in the adipose tissues of rats. Atherosclerosis (oxidized LDL‐C inside the walls of arteries) and obesity have both been linked to the buildup of LDL‐C and TGs (Cai et al., 2015). In support of this, Gao et al. (2017) found that BPA has a hyperlipidemic and compounding effect in the induction of oxidative stress in serum and adipose tissues in rats, which leads to the generation of reactive oxygen species (ROS) such as the hydroxyl radical, peroxynitrite anion, nitric oxide, singlet oxygen, and the peroxyl radical, confirming its deleterious effect in this study.

The decrease in HDL‐C levels observed in this study may be brought on by toxicant and metabolite binding to the enzyme's active site, which suppresses the synthesis of 3‐hydroxy‐3‐methylglutaryl‐coenzyme‐A reductase (HMG‐CoA reductase) and prevents it from catalyzing the conversion of 3‐hydroxy‐3‐methylglutaryl‐coenzyme‐A to mevalonic acid, a critical step in biosynthesis (Naomi et al., 2022).

On the other hand, coadministration of BPA and CMSO substantially decreased cholesterol, triglycerides, and LDL‐C while increasing HDL‐C. It has been reported that the antioxidant and chemical components in CMSO, including omega‐6 fatty acids, omega‐3 fatty acids, palmitic acids, stearic acids, oleic acids, linoleic acids, and other monounsaturated and polyunsaturated fatty acids, protect the structural and functional integrity of the cell membrane and maintain HDL‐C (Boubekeur et al., 2022; Zahnit et al., 2022). These components may also be responsible for the decrease in cholesterol, triglycerides, and LDL‐C. As a result, when hormones indicate a need for energy, fatty acids and glycerol are released from triglycerides stored in fat cells (adipocytes) and transported to organs and tissues throughout the body, or fatty acids are released from adipocytes and mobilized for usage during times of stress (Egbuna et al., 2021; Iwaniec et al., 2007). Fatty acids are attached to a protein called serum albumin in the blood; they are taken up by cells in muscle tissue and oxidized to CO_2_ and water to provide energy (Gao et al., 2017). A study reported that 50 μg/kg/day BPA exposure to 15 weeks old female Sprague–Dawley rats via oral gavage on the 6th day after pregnancy up to 36 days elevated the level of abdominal lipid weight up to 77% in female offspring, decreased level of HDL up to 49% and increased level of TG, TC, LDL, and leptin.

Interestingly, BPA treatment significantly (*p* < .05) increased serum and adipose tissue levels of leptin while concurrently reducing levels of adiponectin. The findings of this study matched those of Angle et al. (2013), who found a significant rise in serum leptin levels with a concomitant drop in serum adiponectin levels, resulting in abdominal fat mass. Similarly, Gao and Horvath (2008) found that BPA induction increased the level of leptin in adipose tissues, which affects body weight by acting primarily on the central nervous system, specifically the hypothalamus, with concomitant decreases in serum levels of adiponectin, which plays a key role in the regulation of glucose uptake by cells (Ben‐Jonathan et al., 2009).

In an in vitro investigation employing human adipocytes, Valentino et al. (2013) discovered that modest doses of BPA affected insulin‐stimulated glucose utilization and the insulin signaling pathway, thus dysregulating adipocyte activity. Additionally, they discovered that male offspring had higher levels of insulin and leptin and lower levels of serum adiponectin, indicating that BPA exposure affects how white adipose tissue is metabolized (Bouret et al., 2015). According to a previous study (Cirillo et al., 2008), BPA has been shown to increase the level of leptin in adipose tissues, which promotes obesity and inflammatory disorders like hypertension, metabolic syndrome, and cardiovascular disease. Leptin bioaccumulation occurs when genes that regulate its activities are modulated, resulting in an increase in leptin levels in the blood (Cirillo et al., 2008).

As a result, when hormones signal a need for energy, fatty acids and glycerol are released from triglycerides stored in fat cells (adipocytes) and delivered to organs and tissues throughout the body, or fatty acids are released from adipocytes and mobilized for use during times of stress when the body requires energy (Iwaniec et al., 2007). The process starts when blood levels of glucagon and adrenaline rise, and these hormones connect to particular receptors on adipose cell surfaces (Goliasch et al., 2015). The activation of lipase, which hydrolyzes triglycerides in the droplet to release free fatty acids, is the result of this binding action in the cell (Cai et al., 2015). These fatty acids enter the circulatory system and are distributed to skeletal and cardiac muscle, as well as liver cells (Iwaniec et al., 2007). Fatty acids are attached to serum albumin in the blood, taken up by the cells, and oxidized to CO_2_ and water to provide energy (Gao et al., 2017). A considerable portion of the fatty acids is taken up by the liver and are partially resynthesized into triglycerides and delivered to the muscle and other tissues in VLDL lipoproteins (Iwaniec et al., 2007). A portion is also transformed into tiny ketone molecules, which are then transported to peripheral organs and consumed to produce energy (Sirtori, 2015).

The bisphenol A (BPA) injection increased the coronary risk index and atherogenic index (AI) in the serum and adipose tissue of male rats considerably (*p* < .05). In an earlier study (Bouret et al., 2015), it was discovered that BPA induction raised the atherogenic index (AI) and coronary risk index (CRI) in rats' serum and adipose tissues, both of which are important biomarkers for determining the likelihood of developing coronary artery disease (CAD).

This finding coincided with an animal study conducted in the United State of America, which found that BPA injection in rats increased AI and CRI in serum and adipose tissues above normal ranges and was inversely associated with BMI (Iwaniec et al., 2007). Similarly, other studies reported that BPA induction in rats inversely increased AI in both serum and adipose tissues of male rats, which eventually leads to cardiovascular diseases such as atherosclerosis and arteriosclerosis thickening or hardening the loss of elasticity of artery walls, restricting blood flow to one's organs and tissues and posing serious health risks.

The findings of this work are comparable to the study of Wang et al. (2020) who looked at the effects of BPA in rats and combined two lipid indices (TAGs and HDL‐C) to create AI, which can be used as a new and better biomarker for obesity. AI is a biomarker of plasma atherosclerosis and it is substantially linked with other major atherosclerosis indexes such as LDL‐C size and small‐dense LDL‐C (Knight et al., 2009). According to Zhu et al. (2015), the accumulation of metabolites and toxicants binding to the enzyme's active site lowers lipase activity and causes the formation of fatty plaques, cholesterol, and other substances in and on the arterial wall, maybe causing an increase in AI and CRI in this study. Contrarily, the coronary risk index and atherogenic index were significantly reduced by BPA and CMSO. The lower levels of AI and CRI in rats' serum and adipose tissues may be attributed to CMSO's previously identified antioxidant and chemical components.

## 5 CONCLUSION

The results of this study showed that BPA exposure in rats increased serum and adipose tissue levels of cholesterol, triglycerides, LDL‐C, leptin, coronary risk index, atherogenic index, and body weight with decreased HDL‐C, and adiponectin. Interestingly, CMSO treatment reduced the toxicity caused by BPA in rats by modulating the body weight, adiponectin and leptin levels, and lipid profiles in serum and adipose tissue. The findings of this study demonstrate CMSO's therapeutic potential capable of alleviating BPA‐induced dyslipidemia and adipokine dysfunction in male rats. However, as sufficient studies have not been done on *Cucumeropsis mannii* seed oil, we recommend further studies into the biological activities of the plant oil and also establish more information on the specific compounds responsible for each biological activity.

## CONFLICT OF INTEREST STATEMENT

The authors declare no conflict of interest.

## ETHICS STATEMENT

This study received ethical approval number EBSU/BCH/ET/20/003 from the Ethical Committee of the Biochemistry Department, EBSU, and followed their recommendations.

## CONSENT FOR PUBLICATION

All the authors of this manuscript gave their consent to publish.

## Data Availability

Additional data will be made available on request.

## References

[fsn33271-bib-0001] Abbott, R. D. , Wilson, P. W. , Kannel, W. B. , & Casteli, W. P. (1998). High density lipoprotein cholesterol, total cholesterol screening and myocardial infraction. The Framingham study. Arteriosclerosis,Thrombosis, and Vascular Biology, 8, 207–211. 10.1161/01.atv.8.3.207 3370018

[fsn33271-bib-0002] Adeghe, O. M. , & Emejulu, M. J. (2016). Evaluation of bisphenol A, physicochemical properties and microbial characterization of borehole water stored in plastic containers. Journal of Applied Science and Environmental Management, 20(4), 1119–1124.

[fsn33271-bib-0003] Agu, P. C. , Aja, P. M. , Ugbala, E. E. , Ogwoni, H. A. , Ezeh, E. M. , Oscar‐Amobi, P. C. , Atamgba, A. A. , Ani, O. G. , Awoke, J. N. , Nwite, F. E. , Ukachi, O. U. , Orji, O. U. , Nweke, P. C. , Ugbala, E. E. , Ewa, G. O. , Igwenyi, I. O. , Egwu, C. O. , Alum, E. U. , Chukwu, D. C. , & Famurewa, A. C. (2022). *Cucumeropsis mannii* seed oil (CMSO) attenuates alterations in testicular biochemistry and histology against Bisphenol a‐induced toxicity in male Wister albino rats. Heliyon, 8(2022), e09162. 10.1016/j.heliyon.2022.e09162 35846473 PMC9280550

[fsn33271-bib-0004] Akash, S. M. H. , Sabir, S. , & Rehman, K. (2020). Bisphenol A‐induced metabolic disorders: From exposure to mechanism of action. Environmental Toxicology and Pharmacology, 77, 1–13. 10.1016/j.etap.2020.103373 32200274

[fsn33271-bib-0005] Alboghobeish, S. , Mahdavinia, M. , Zeidooni, L. , Samimi, A. , Oroojan, A. A. , Alizadeh, S. , Dehghani, M. A. , Ahangarpour, A. , & Khorsandi, L. (2019). Efficacy of naringin against reproductive toxicity and testicular damages induced by bisphenol A in rats. Iranian Journal of Basic Medical Science, 22, 315–323. 10.22038/ijbms.2019.29757.7184 PMC6528718 31156794

[fsn33271-bib-0006] Alladi, S. , & Shanmugasundaram, K. R. (1989). Induction of hypercholesterolemia by supplementing soy protein with acetate generating amino‐acids. Nutritional Report International, 40, 893–899.

[fsn33271-bib-0007] Allain, O. P. , & Roschlaw, A. P. (1979). Biochemical analysis (3rd ed., pp. 80–89). Oxford University Press.

[fsn33271-bib-0008] Amin, M. M. , Ebrahim, K. , Hashemi, M. , Rafiei, N. , Mansourian, M. , & Kelishadi, R. (2018). Association of exposure to Bisphenol A with obesity and cardiometabolic risk factors in children and adolescents. International Journal of Environmental Health Research, 29, 94–106. 30203985 10.1080/09603123.2018.1515896

[fsn33271-bib-0009] Angle, B. M. , Do, R. P. , Ponzi, D. , & Stahlhut, R. W. (2013). Metabolic disruption in male adipokine release from human adipose tissue: Implications for the metabolic syndrome. Molecular Cell Endocrinology, 304(2), 49–54. 10.1016/j.mce.2009.02.022 PMC2775425 19433247

[fsn33271-bib-0010] Apau, J. , Acheampong, A. , & Adua, E. (2018). Exposure to Bisphenol A, Bisphenol F, and Bisphenol S can result in obesity in human body. Cogent Chemistry, 4, 1506601.

[fsn33271-bib-0011] Ariemma, F. , Esposito, V. D. , Liguoro, D. , Oriente, F. , Beguinot, F. , Formisano, P. , & Valentino, R. (2016). Low‐dose Bisphenol‐a impairs adipogenesis and generates dysfunctional 3T3‐L1 adipocytes. PLoS One, 11(3), e0150762. 26942597 10.1371/journal.pone.0150762 PMC4778877

[fsn33271-bib-0012] Awuchi, C. G. , & Awuchi, C. G. (2019). Physiological effects of plastic wastes on the endocrine system (Bisphenol A, phthalates, Bisphenol S, PBDEs, TBBPA). International Journal of Bioinformatics and Computational Biology, 4(2), 11–29.

[fsn33271-bib-0013] Ben‐jonathan, N. , Hugo, E. R. , & Brandebourg, T. D. (2009). Molecular and cellular endocrinology effects of bisphenol A on adipokine release from human adipose tissue. Molecular and Cellular Endocrinology, 304, 49–54. 19433247 10.1016/j.mce.2009.02.022 PMC2775425

[fsn33271-bib-0014] Besong, S. A. , Ezekwe, M. O. , Fosung, C. N. , & Senwo, Z. N. (2011). Evaluation of nutrient composition of African melon oilseed (*Cucumeropsis mannii* Naudin) for human nutrition. International Journal of Nutrition and Metabolism, 3(8), 103–108.

[fsn33271-bib-0015] Boubekeur, S. , Messaoudi, M. , Awuchi, C. G. , Otekunrin, O. , Sawicka, B. , Idjeri‐Mecherara, S. , Bouchareb, S. , Hassani, A. , Sharifi‐Rad, M. , Begaa, S. , & Rebiai, A. (2022). Biological properties and polyphenols content of Algerian *Cistus salviifolius* L. aerial parts. European Journal of Biological Research, 12(2), 163–180. 10.5281/zenodo.6561505

[fsn33271-bib-0016] Bouret, S. , Levin, B. E. , & Ozanne, S. E. (2015). Gene‐environment interactions controlling energy and glucose homeostasis and the developmental origins of obesity. Physiological Reviews, 95(1), 47–82. 25540138 10.1152/physrev.00007.2014 PMC4281588

[fsn33271-bib-0017] Cai, G. , Zhang, B. , & Shi, G. (2015). The associations between proprotein convertase subtilisin/Kexin type 9 E670G polymorphism and the risk of coronary artery disease and serum lipid levels: A meta‐analysis. Lipids Health Disease, 14, 149. 10.1186/s12944-015-0154-7 PMC4650262 26576960

[fsn33271-bib-0018] Cirillo, D. , Rachiglio, A. M. , Montagna, R. , & Giordano, A. (2008). Leptin signaling in breast cancer. Journal of Cellular Biochemistry, 105(4), 956–964. 18821585 10.1002/jcb.21911

[fsn33271-bib-0019] Colucci, G. , Santamaria‐Echart, A. , Silva, S. C. , Fernandes, I. P. M. , Sipoli, C. C. , & Barreiro, M. F. (2020). Development of water‐in‐oil emulsions as delivery vehicles and testing with a natural antimicrobial extract. Molecules (Basel, Switzerland), 25(9), 2105. 10.3390/molecules25092105 32365923 PMC7248747

[fsn33271-bib-0020] Desai, M. , Ferrini, M. G. , Jellyman, J. K. , Han, G. , & Ross, M. G. (2018). In vivo and in vitro Bisphenol A exposure effects on adiposity. Journal of Developmental Origins of Health and Disease, 9, 678–687. 10.1017/S2040174418000600 30156179 PMC6363869

[fsn33271-bib-0021] Dhakad, P. K. , Sharma, P. K. , & Kumar, S. (2017). A review on phytochemical studies and biological potential of *Cucumeropsis mannii seed* (L.) Schrad. (Cucurbitaceae). Bioengineering and Bioscience, 5, 55–64.

[fsn33271-bib-0022] Dror, Y. , Cohen, Y. , & Yerushalmi‐Rozen, R. (2006). Structure of gum Arabic in aqueous solution. Journal of Polymer Science Part B: Polymer Physics, 44(22), 3265–3271.

[fsn33271-bib-0023] Egbuna, C. , Awuchi, C. G. , Kushwaha, G. , Rudrapal, M. , Patrick‐Iwuanyanwu, K. C. , Singh, O. , Odoh, U. E. , Khan, J. , Jeevanandam, J. , Kumarasamy, S. , Chukwube, V. O. , Narayanan, M. , Palai, S. , Găman, M. A. , Uche, C. Z. , Ogaji, D. S. , Ezeofor, N. J. , Mtewa, A. G. , Patrick‐Iwuanyanwu, C. C. , … Chikwendu, C. J. (2021). Bioactive compounds effective against type 2 diabetes mellitus: A systematic review. Current Topics in Medicinal Chemistry, 21(12), 1067–1095. 10.2174/1568026621666210509161059 33966619

[fsn33271-bib-0024] Eleazu, K. , Aja, P. M. , & Eleazu, C. O. (2021). Cocoyam (*Colocasia esculenta*) modulates some parameters of testosterone propionate induced rat model of benign prostatic hyperplasia. Drug and Chemical Toxicology, 45(5), 1923–1933. 33641553 10.1080/01480545.2021.1892956

[fsn33271-bib-0025] Eleazu, K. , Maduabuchi, P. , & Eleazu, C. (2018). Effect of ethanol extract of boiled breadfruit (Treculia Africana) seed on the oral glucose tolerance, lipid profile, and body weight of normoglycemic albino rats. Wiley Food Science and Nutrition, 6, 904–911. 10.1002/fsn3.626 PMC6021728 29983953

[fsn33271-bib-0026] Flint, S. , Markle, T. , Thompson, S. , & Wallace, E. (2012). Bisphenol A exposure, effects, and policy: A wildlife perspective. Journal of Environmental Management, 104, 19–34. 22481365 10.1016/j.jenvman.2012.03.021

[fsn33271-bib-0027] Friedewald, W. T. , Levy, R. I. , & Fredrickson, D. S. (1972). Estimation of the concentration of low‐density lipoprotein cholesterol in plasma, without the use of the preparative ultracentrifuge. Clinical Chemistry, 18, 499–502. 4337382

[fsn33271-bib-0028] Gabr, A. A. , Mahfouz, N. N. , Shady, M. M. A. , Youssef, M. M. , El‐din, E. M. S. , Kamhawy, A. H. , Hussein, J. , Ibrahim, T. S. , & Abbas, M. A. (2017). Socioeconomic position as a risk factor for BPA exposure in a sample of Egyptian children. Journal of Applied Pharmaceutical Science, 7(12), 84–89.

[fsn33271-bib-0029] Gao, M. , Zheng, Y. , & Zhang, W. (2017). Non‐high‐density lipoprotein cholesterol predicts nonfatal recurrent myocardial infarction in patients with segment elevation myocardial infarction. Lipids Health Disease, 16, 20. 10.1186/s12944-017-0418-5 PMC5260128 28114933

[fsn33271-bib-0030] Gao, Q. , & Horvath, T. L. (2008). Cross‐talk between estrogen and leptin signaling in the hypothalamus. Journal of Physiology, Endocrinology, and Metabolism, 294(5), 817–826. 10.1152/ajpendo.00733.2007 18334610

[fsn33271-bib-0031] Gebreegziabiher, G. , Belachew, T. , Mehari, K. , & Tamiru, D. (2021). Prevalence of dyslipidemia and associated risk factors among adult residents of Mekelle City, Northern Ethiopia. PloS One, 16(2), e0243103. 10.1371/journal.pone.0243103 33561153 PMC7872241

[fsn33271-bib-0032] Goliasch, G. , Wiesbauer, F. , & Blessberger, H. (2015). Premature myocardial infarction is strongly associated with increased levels of remnant cholesterol. Journal Clinical Lipid, 9, 801–896. 10.1016/j.jacl.2015.08.009 26687701

[fsn33271-bib-0033] Gurmeet, K. S. S. , Rosnah, I. , Normadiah, M. K. , Das, S. , & Mustafa, A. M. (2014). Detrimental effects of Bisphenol A on development and functions of the male reproductive system in experimental rats. EXCLI Journal of Experimental and Clinical Science, 13, 151–160. PMC4464354 26417249

[fsn33271-bib-0034] Hotta, K. , Funahashi, T. , Arita, Y. , Takahashi, M. , Matsuda, M. , Okamoto, Y. , Iwahashi, H. , Kuriyama, H. , Ouchi, N. , Maeda, K. , Nishida, M. , Kihara, S. , Sakai, N. , Nakajima, T. , Hasegawa, K. , Muraguchi, M. , Ohmoto, Y. , Nakamura, T. , Yamashita, S. , … Matsuzawa, Y. (2000). Plasma concentrations of a novel, adipose‐specific protein, adiponectin, in type 2 diabetic patients. Arterioscleresosis Thrombosis and Vascular Biology, 20(6), 1595–1599. 10.1161/01.atv.20.6.1595 10845877

[fsn33271-bib-0035] Iwaniec, U. T. , Boghossian, S. , Lapke, P. D. , Turner, R. T. , & Kalra, S. P. (2007). Central leptin gene therapy corrects skeletal abnormalities in Leptin‐deficient mice. Peptides, 28(5), 1012–1912. 17346852 10.1016/j.peptides.2007.02.001 PMC1986832

[fsn33271-bib-0036] Kate, A. E. , Lohani, U. C. , Pandey, J. P. , Shahi, N. C. , & Sarkar, A. (2014). Traditional and mechanical method of the oil extraction from wild apricot kernel: A comparative study. Research Journal of Chemical and Environmental Science, 2(2), 54–60.

[fsn33271-bib-0037] Khalid, W. , Arshad, M. S. , Aslam, N. , Mukhtar, S. , Rahim, M. A. , Ranjha, M. M. A. N. , Noreen, S. , Afzal, M. F. , Aziz, A. , & Awuchi, C. G. (2022). Food applications of sorghum derived kafirins potentially valuable in celiac disease. International Journal of Food Properties, 25(1), 2348–2363. 10.1080/10942912.2022.2135532

[fsn33271-bib-0038] Kim, J. A. , & Choi, K. M. (2020). Newly discovered adipokines: Pathophysiological link between obesity and Cardiometabolic disorders. Frontiers in Physiology, 11, 568800. 10.3389/fphys.2020.568800 32982804 PMC7492654

[fsn33271-bib-0039] Knight, W. D. , Seth, R. , Boron, J. , & Overton, J. M. (2009). Short‐term physiological hyperleptinemia decreases arterial blood pressure. Regulation Peptide, 154(3), 60–78. 10.1016/j.regpep.2009.02.001 19323984

[fsn33271-bib-0040] Messaoudi, M. , Rebiai, A. , Sawicka, B. , Atanassova, M. , Ouakouak, H. , Larkem, I. , Egbuna, C. , Awuchi, C. G. , Boubekeur, S. , Ferhat, M. A. , Begaa, S. , & Benchikha, N. (2022). Effect of extraction methods on polyphenols, flavonoids, mineral elements, and biological activities of essential oil and extracts of *Mentha pulegium* L. Molecules, 27(1), 11. 10.3390/molecules27010011 PMC8746320 35011242

[fsn33271-bib-0041] Metz, C. M. (2016). Bisphenol A: Understanding the controversy. Workplace Health and Safety, 64(1), 28–36. 26800896 10.1177/2165079915623790

[fsn33271-bib-0042] Miao, M. , Yuan, W. , & Yang, F. (2015). Associations between bisphenol‐a exposure and reproductive hormones among female workers. International Journal of Environmental Research and Public Health, 10, 13240–13250. 10.3390/ijerph121013240 PMC4627028 26506366

[fsn33271-bib-0043] Müllerová, D. , & Kopecký, J. (2007). White adipose tissue: Storage and effector site for environmental pollutants. Physiological Research, 56(4), 375–381. 16925464 10.33549/physiolres.931022

[fsn33271-bib-0044] Nanjappa, M. K. , Simon, L. , & Akingbemi, B. T. (2012). The industrial chemical Bisphenol A (BPA) interferes with proliferative activity and the development of steroidogenic capacity in rats leydig cells. Biology of Reproduction, 86(5), 135. 22302688 10.1095/biolreprod.111.095349 PMC3364919

[fsn33271-bib-0045] Naomi, R. , Yazid, M. D. , Bahari, H. , Keong, Y. Y. , Rajandram, R. , Embong, H. , Teoh, S. H. , Halim, S. , & Othman, F. (2022). Bisphenol A (BPA) leading to obesity and cardiovascular complications: A compilation of current In vivo study. International Journal of Molecular Sciences, 23, 2969. 10.3390/ijms23062969 35328389 PMC8949383

[fsn33271-bib-0046] Neier, K. , Marchlewicz, E. M. , Bedrosian, L. D. , Dolinoy, D. C. , & Harris, C. (2019). Characterization of the mouse white adipose tissue redox environment and associations with perinatal environmental exposures to bisphenol A and high‐fat diets. The Journal of Nutritional Biochemistry, 66, 86–97. 30776609 10.1016/j.jnutbio.2019.01.005 PMC7003727

[fsn33271-bib-0047] Nelson, D. L. , & Cox, M. M. (2008). Lehninger principles of biochemistry (5th ed.). W. H. Freeman and Company.

[fsn33271-bib-0048] Norazit, A. , Mohamad, J. , Razak, S. A. , Abdulla, M. A. , & Azmil, A. (2012). Effects of soya bean extract bisphenol a and 17β‐estradiol on the testis and circulating levels of testosterone and estradiol among peripubertal juvenile male Sprague‐Dawley rats. Sains Malaysiana, 41(1), 63–69.

[fsn33271-bib-0049] Nwozo, O. S. , Effiong, E. M. , Aja, P. M. , & Awuchi, C. G. (2023). Antioxidant, phytochemical, and therapeutic properties of medicinal plants: A review. International Journal of Food Properties, 26(1), 359–388. 10.1080/10942912.2022.215742

[fsn33271-bib-0050] OECD . (2008). Acute oral toxicity testing procedures. http://www.oecd.org/env/testguidelines

[fsn33271-bib-0051] Ofoedu, C. E. , Ofoedu, E. O. , Chacha, J. S. , Owuamanam, C. I. , Efekalam, I. S. , & Awuchi, C. G. (2022). Comparative evaluation of physicochemical, antioxidant, and sensory properties of red wine as markers of its quality and authenticity. International Journal of Food Science, 2022, 8368992. 10.1155/2022/8368992 36299559 PMC9592215

[fsn33271-bib-0052] Rahim, M. A. , Naeem, M. , Khalid, K. , Imran, M. , Khan, M. K. , Khan, M. I. , Nisa, M. U. , Sarwar, M. , & Awuchi, C. G. (2023). Effects of different levels of egg protein replacement in weaned diets on hematology, kidney functions, and immunity biomarkers. Food Science & Nutrition, 11, 1–8. 10.1002/fsn3.3204 PMC10084960 37051337

[fsn33271-bib-0053] Rahim, M. A. , Umar, M. , Habib, A. , Imran, M. , Khalid, W. , Lima, C. M. G. , Shoukat, A. , Itrat, N. , Nazir, A. , Ejaz, A. , Zafar, A. , Awuchi, C. G. , Sharma, R. , Santana, R. F. , & Emran, T. B. (2022). Photochemistry, functional properties, food applications, and health prospective of black rice. Journal of Chemistry, 2022, 2755084. 10.1155/2022/2755084

[fsn33271-bib-0054] Rönn, M. , Lind, L. , Örberg, J. , Kullberg, J. , Söderberg, S. , Larsson, A. , Johansson, L. , Ahlström, H. , & Lind, P. M. (2014). Bisphenol A is related to circulating levels of adiponectin, leptin and ghrelin, but not to fat mass or fat distribution in humans. Chemosphere, 112, 42–48. 25048886 10.1016/j.chemosphere.2014.03.042

[fsn33271-bib-0055] Sirtori, C. R. (2015). HDL and the progression of atherosclerosis: New insights. European Heart Journal Supplements, 2, 33–37.

[fsn33271-bib-0056] Stragierowicz, J. (2015). Bisphenol a – Application, sources of exposure and potential risks in infants, children and pregnant women. International Journal of Occupational Medicine and Environmental Health, 28, 209–241. 26182919 10.13075/ijomeh.1896.00343

[fsn33271-bib-0058] Talpade, J. , Shrman, K. , Sharma, R. K. , Gutham, V. , Singh, R. P. , & Meena, N. S. (2018). Bisphenol A: An endocrine disruptor. Journal of Entomology and Zoology Studies, 6(3), 394–397.

[fsn33271-bib-0059] Taylor, J. A. , Shioda, K. , Mitsunaga, S. , Yawata, S. , Angle, B. M. , Nagel, S. C. , vom Saal, F. S. , & Shioda, T. (2018). Prenatal exposure to bisphenol A disrupts naturally occurring bimodal dna methylation at proximal promoter of fggy, an obesity‐relevant gene encoding a carbohydrate kinase, in gonadal white adipose tissues of CD‐1 mice. Endocrinology, 159, 779–794. 29220483 10.1210/en.2017-00711 PMC5774244

[fsn33271-bib-0060] Tian, S. , Yan, S. , Meng, Z. , Huang, S. , Sun, W. , Jia, M. , Teng, M. , Zhou, Z. , & Zhu, W. (2021). New insights into bisphenols induced obesity in zebrafish (*Danio rerio*): Activation of cannabinoid receptor CB1. Journal of Hazardous Materials, 418, 126100. 34098260 10.1016/j.jhazmat.2021.126100

[fsn33271-bib-0061] Tietz, N. W. (1990). Serum triglyceride determination: Clinical guide to laboratory tests (pp. 554–556). Saunders Co.

[fsn33271-bib-0062] Tufail, T. , Ijaz, A. , Noreen, S. , Arshad, M. U. , Gilani, S. A. , Bashir, S. , Din, A. , Shahid, M. Z. , Khan, A. A. , Khalil, A. A. , & Awuchi, C. G. (2021). Pathophysiology of obesity and diabetes. In C. Egbuna & S. Hassan (Eds.), Dietary phytochemicals (pp. 29–42). Springer. 10.1007/978-3-030-72999-8_2

[fsn33271-bib-0063] Tusubira, D. , Aja, P. M. , Munezero, J. , Ssedyabane, F. , Namale, N. , Ifie, J. E. , Agu, P. C. , Ajayi, C. O. , & Okoboi, J. Safety profile of Colocasia esculenta tuber extracts in benign prostate hyperplasia. 30 August 2022, PREPRINT (Version 1) available at Research Square. 10.21203/rs.3.rs-1930275/v1 PMC10249314 37286957

[fsn33271-bib-0064] Valentino, R. , Esposito, V. , & Passaretti, F. (2013). Bisphenol A impairs insulin action and up‐regulates inflammatory pathways in human subcutaneous adipocytes. Journal of Pone, 8(12), 82–99. 10.1371/journal.pone.0082099 PMC3857211 24349194

[fsn33271-bib-0065] Wang, B. , Wang, S. , Zhao, Z. , Chen, Y. , Xu, Y. , Li, M. , Xu, M. , Wang, W. , Ning, G. , Bi, Y. , & Wang, T. (2020). Bisphenol A exposure in relation to altered lipid profile and dyslipidemia among Chinese adults: A repeated measures study. Environmental Research, 184, 109382. 10.1016/j.envres.2020.109382 32192991

[fsn33271-bib-0066] Watal, G. , Yadav, M. , Chatterji, S. , & Gupta, S. K. (2014). Preliminary phytochemical screening of six medicinal plants used in traditional medicine. International Journal of Pharmacy and Pharmaceutical Sciences, 6(5), 539–542.

[fsn33271-bib-0067] Yasmin, I. , Khan, W. A. , Naz, S. , Iqbal, M. W. , Awuchi, C. G. , Egbuna, C. , Hassan, S. , Patrick‐Iwuanyanwu, K. C. , & Uche, C. Z. (2021). Etiology of obesity, cancer, and diabetes. In C. Egbuna & S. Hassan (Eds.), Dietary phytochemicals (pp. 1–27). Springer. 10.1007/978-3-030-72999-8_1

[fsn33271-bib-0068] Zahnit, W. , Smara, O. , Bechki, L. , Souici, C. B. , Messaoudi, M. , Benchikha, N. , Larkem, I. , Awuchi, C. G. , Sawicka, B. , & Simal‐Gandara, J. (2022). Phytochemical profiling, mineral elements, and biological activities of *Artemisia campestris* L. grown in Algeria. Horticulturae, 8(10), 914. 10.3390/horticulturae8100914

[fsn33271-bib-0069] Zheng, J. , Xiao, Z. , Zhang, K. , Qiu, X. , Luo, L. , & Li, L. (2020). Improved blind tracheal intubation in rats: A simple and secure approach. The Journal of Veterinary Medical Science, 82(9), 1329–1333. 10.1292/jvms.20-0267 32741885 PMC7538325

[fsn33271-bib-0070] Zhu, L. , Lu, Z. , & Zhu, K. (2015). Lipoprotein ratios are better than conventional lipid parameters in predicting coronary heart disease in Chinese Han people. Cardiology, 73, 931–998. 10.5603/KP.a2015.0086 25985729

